# MULTILEVEL ANALYSIS OF AARC GAINS AND LOSSES IN OLDER ADULTS

**DOI:** 10.1093/geroni/igae098.4026

**Published:** 2024-12-31

**Authors:** Natsue Shiraishi, Yasuhiro Yamamoto, Takeshi Nakagawa, Takashi Horiuchi

**Affiliations:** Okayama University, Okayama, Okayama, Japan; Hyogo University of Teacher Education, Kato, Hyogo, Japan; Osaka University, Suita, Osaka, Japan; Okayama University, Okayama, Okayama, Japan

## Abstract

This study investigates the factors associated with Awareness of Age-Related Change (AARC) gains and losses in older adults. Numerous educational institutions offer programs tailored to older adults, fostering continuous learning. Therefore, in the case of older people, lifelong learning, and not just educational history, should be investigated. Thus, this study examined whether lifelong learning as social participation is associated with AARC. We conducted a comprehensive analysis using an 12-month 3-wave survey collected between 2023 and 2024 (N=878, ages 70 and above at baseline). The independent variables were those measured at Time 1. AARC was analyzed in association with initial values. As a result, initial AARC-gain values were higher for those with more lifetime learning at Time 1. Our multilevel model results indicate that age (b = 0.02, p < 0.001) and lifelong learning (b = 0.11, p < 0.001) significantly are associated with levels of AARC-gains, while physical activity (b = -0.0002, p < 0.001) and IADL (b = -1.49, p < 0.001) are associated with levels of AARC-losses. No individual differences in slope were observed. These findings underscore the importance of continuous education and physical activity in mitigating age-related losses and enhancing gains, providing valuable insights for interventions aimed at improving the well-being of older adults.

